# Characterization of an Immortalized Human Microglial Cell Line as a Tool for the Study of Diabetic Retinopathy

**DOI:** 10.3390/ijms23105745

**Published:** 2022-05-20

**Authors:** Aurora Mazzeo, Massimo Porta, Elena Beltramo

**Affiliations:** Department of Medical Sciences, University of Torino, 10126 Torino, Italy; aurora.mazzeo@unito.it (A.M.); massimo.porta@unito.it (M.P.)

**Keywords:** diabetes, diabetic retinopathy, human microglia, inflammation, neurovascular unit, retina

## Abstract

The complexity of the retinal structure reflects on the difficulty to describe its composite cell interactions. Microglia is responsible for the immune reaction to inflammatory stimuli during diabetic retinopathy (DR), but most studies still use rodent cells. We characterized a commercially available immortalized human microglial line and tested its susceptibility to inflammation, to study the interactions between the neuro-vascular retinal portions in species-specific models. After checking the expression of microglial markers, we tried lipopolysaccharide (LPS) stimulation and several pro-inflammatory cocktails to select the best combination able to induce a significant M1 (inflammatory) response. We measured M1 induction through the expression of pro- and anti-inflammatory molecules and performed morphologic and functional assays. Marker expression confirmed the human microglial derivation of these cells. Differently from rodents, LPS did not induce a M1 profile. The best pro-inflammatory stimulus was an interleukin-1β + tumor necrosis factor-α + interferon-γ cocktail, which induced morphology changes and increased proliferation, apoptosis, migration, reactive oxygen species, and the expression of inflammatory cytokines and miRNAs. In conclusion, this microglial line proved potentially useful to investigate the cascade of events leading to DR. In perspective, co-culture models involving microvascular cells will help in the understanding of multifaceted interactions of the neurovascular unit.

## 1. Introduction

The complexity of the retinal structure, with its several layers and cell types, reflects on the difficulty to describe the composite cell interactions, as well as the pathways involved in maintaining health. This scenario becomes even more complex in pathological conditions affecting the retina. As regards diabetic retinopathy (DR), awareness is increasing that pathogenic mechanisms involved in this microvascular complication are greatly articulated and dynamic. Involvement of the neuroretina since the earlier stages of the disease is now acknowledged, and DR is no longer considered a purely microvascular disorder [1,2]. Endothelial cells, pericytes, and microglia are all involved in a complex interplay.

Pericytes and endothelial cells are fundamental components of the central nervous system (CNS) microvasculature, regulating the blood–brain and blood–retinal barriers, vessel formation, stabilization, and remodeling [3]. Microglia is made up of myeloid cells, which are key players of the inflammatory stimuli [4]. In the CNS, as well as in retinal tissue, the microglia constantly monitors the microenvironment [2], responding to damage through the production of immune mediators and aiming to restore tissue homeostasis via the secretion of several factors [5]. Diabetes could be considered a state of chronic inflammation in response to which the microglia becomes activated, switching between two typical states. In the early stages of DR, the microglia swings between M2, its anti-inflammatory state, and M1, its pro-inflammatory phenotype, whereas this delicate balance is impaired as the disease progresses and M1 becomes the prevailing phenotype [2]. The close link between the hyperglycemic milieu characteristic of DR and the activation of microglia, with consequent release of inflammatory factors, is well known. Exposure to high glucose, and especially to the toxic effects of reactive oxygen species (ROS) and advanced glycation-end products (AGE), induces the microglia to switch to the M1 phenotype [6,7,8,9,10]. This, in turn, results in enhanced cell proliferation [11] and increased expression of pro-inflammatory molecules, such as tumor necrosis factor-α (TNF-α), monocyte chemotactic protein-1 (MCP-1, or CCL2) [7], inducible nitric oxide synthase (iNOS) [12], and interleukin-1β (IL-1β) [11]. M1 microglia mediates the release of inflammatory and apoptotic factors by retinal pericytes, while inhibiting the expression of pro-survival molecules [13].

Most studies on retinal inflammation in DR use animal models or rodent microglial lines, because of the scarce availability of human cells. Even though some human microglial cell lines have been established and characterized [14,15,16,17], few studies on them exist, and none, to our knowledge, address DR. We have demonstrated that human retinal pericytes behave very differently from bovine ones when exposed to conditions mimicking the diabetic microenvironment [18,19], and believe that the use of species-specific cell models should constitute the gold standard in in vitro studies.

Consequently, we purchased a new commercially available immortalized human microglial line (IM-HM), carried out a detailed characterization, and tested its susceptibility to inflammatory stimuli, in order to develop a suitable model to study the multiple interactions between the vascular and neuronal components of the retina.

## 2. Results

### 2.1. Morphology

**Figure 1:**
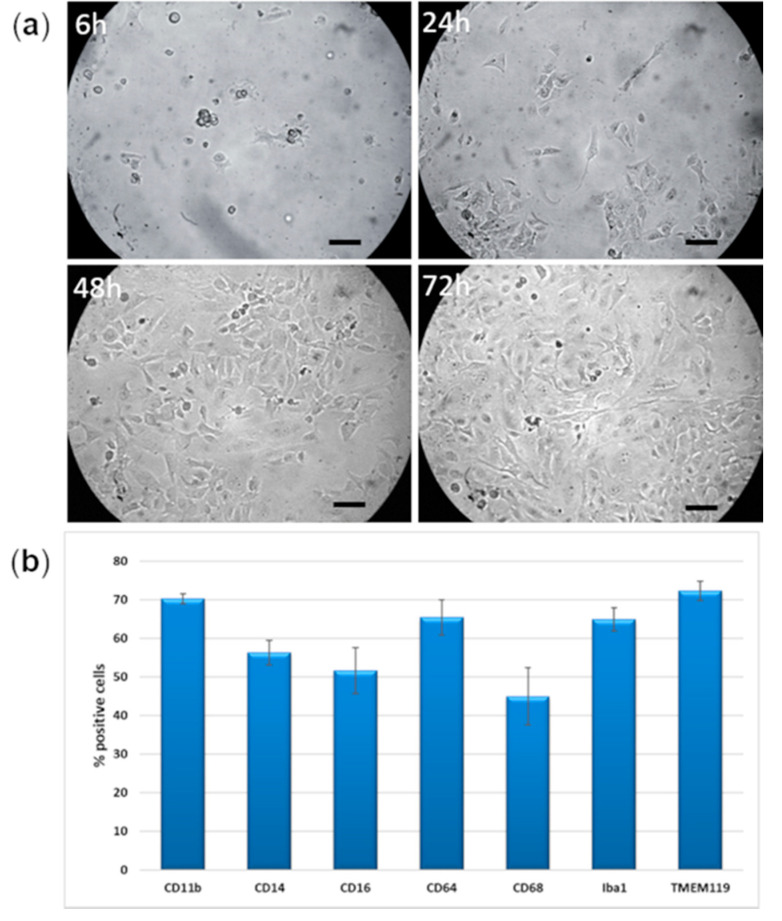
(**a**) Morphological aspect of immortalized human microglial cells (IM-HM) in physiological conditions, after 6, 24, 48, and 72 h from thawing. Images were taken at 20x objective magnification and scale bar indicates 50 μm. (**b**) Expression of human microglial markers in IM-HM in physiological conditions after 48 h from thawing, as a percentage of positive cells.

This microglial line is derived from primary human microglial cells through SV40-immortalization. The manufacturer (Innoprot, Bizkaia, Spain) declares > 99% purity and the maintenance of microglial specific markers, such as Iba1, CD68, TREM2, and CD11b. Cells are shipped in dry ice at a concentration of 1 × 10^6^ cells/vial. In our hands, IM-HM cells, seeded in collagen I pre-coated flasks or plates at a density of about 10,000 cells/cm^2^ in their appropriate medium purchased from the same manufacturer, grow rapidly and reach confluence after about 72 h. They are passaged 1:4/1:5 twice a week. In physiological conditions, they show a characteristic triangular/polygonal shape with several cytoplasmic processes (Figure 1a). These morphological features were maintained at least until passage 15 after purchasing. For the subsequent characterization and experiments, we used IM-HM cells between passages 4–10.

### 2.2. Human Microglia Markers

Figure 1b shows the expression of human microglial markers by flow cytometry analysis, in IM-HM cells cultured in physiological conditions, after 48 h from thawing. CD11b and TMEM119 are expressed in about 70% of IM-HM cells, CD64 and Iba1 around 65%, CD14 and CD16 50–55%, while CD68 is slightly less expressed (45%). 

### 2.3. M1 Activation

We tested several pro-inflammatory factors, single and combined, to find out the best combination to achieve M1 inflammatory activation after 24 h exposure. All factors and combinations are detailed in Table S1.

We first tried different concentrations of lipopolysaccharide (LPS) and times of exposure (1, 2, and 4 days), but could not detect any release of nitric oxide (NO) in the supernatant by Griess reaction. Concurrently, we found no differences in the expression of iNOS, interferon-γ (IFN-γ) (all pro-inflammatory markers) and transforming growth factor-β (TGF-β, anti-inflammatory) when compared with untreated cells.

**Figure 2:**
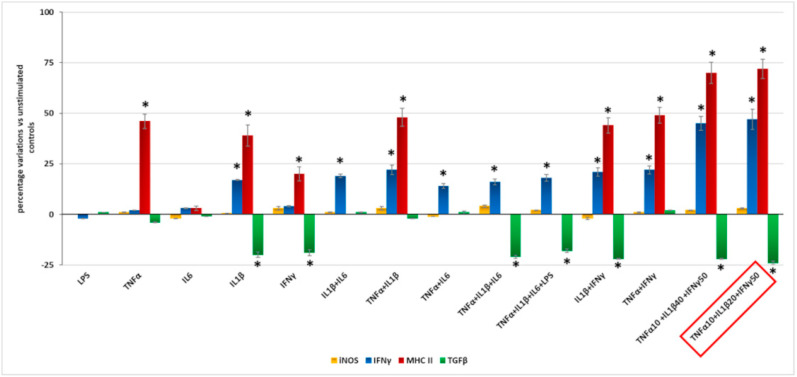
Factors and combinations tested to determine the best mixture to induce M1 activation in microglial cells. All factors are of human origin; concentrations and exposure times were chosen according to the literature. We first measured nitric oxide (NO) release in the supernatants through the Griess reaction, but it was undetectable in all cases (data not shown). Inducible nitric oxide synthase (iNOS), interferon-γ (IFN-γ), MHC I (pro-inflammatory markers), and transforming growth factor-β (TGF-β, anti-inflammatory) were assessed by Western blotting in cell lysates. We selected the combination that exerted the maximum effects with the lowest concentrations: 10 ng/mL tumor necrosis factor-α (TNFα) + 20 ng/mL interleukin-1β (IL1β) + 50 ng/mL IFNγ. Lypopolisaccharide (LPS) had no effect on IM-HM cells. Data are expressed as percentage variations of unstimulated microglial cells. Mean of three experiments ± SD, * *p* < 0.05.

Subsequently, we tested various combinations of TNF-α, IL-1β, interleukin-6 (IL-6), and IFN-γ, and found that they exerted different effects on IFN-γ, MHC class I, and TGF-β, as shown in Figure 2 and Table S1. NO release was undetectable in all supernatants, and no difference in iNOS expression between untreated and treated cells was found in each condition tested. 

For the subsequent experiments we chose the combination that exerted the maximum effects at the lowest concentrations: 10 ng/mL TNF-α + 20 ng/mL IL-1β + 50 ng/mL IFN-γ. 

**Figure 3:**
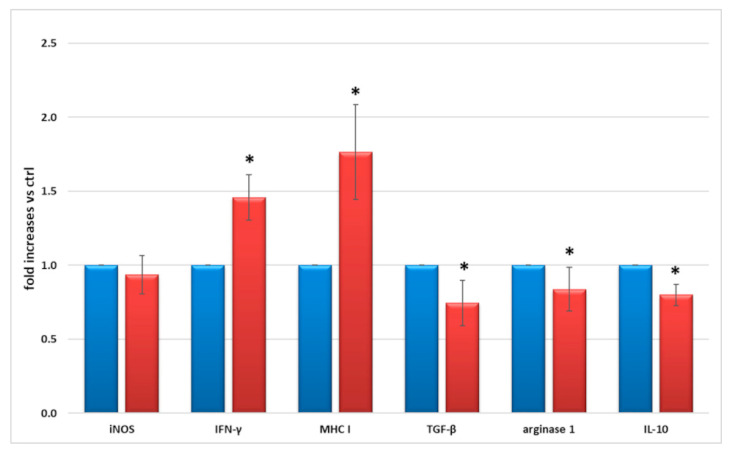
Protein expression of iNOS, IFN-γ, MHC I (pro-inflammatory markers), TGF-β, arginase 1, and IL-10 (anti-inflammatory markers), measured by Western blotting in lysates of cells cultured in control conditions (blue) and after M1 activation (red). M1 activation was achieved by exposing microglial cells for 24 h to TNF-α 10 ng/mL + IL-1β 20 ng/mL + IFN-γ 50 ng/mL. Mean of five experiments ± SD, * *p* < 0.05.

Figure 3 reports a 1.5–1.8-fold increase of pro-inflammatory IFN-γ and MHC class I expression, and a concomitant 20% average decrease of anti-inflammatory TGF-β, arginase 1, and interleukin-10 (IL-10) expression, measured in cell lysates after 24 h M1 exposure, in comparison with control conditions. 

### 2.4. Expression and Secretion of Inflammatory Factors

**Figure 4:**
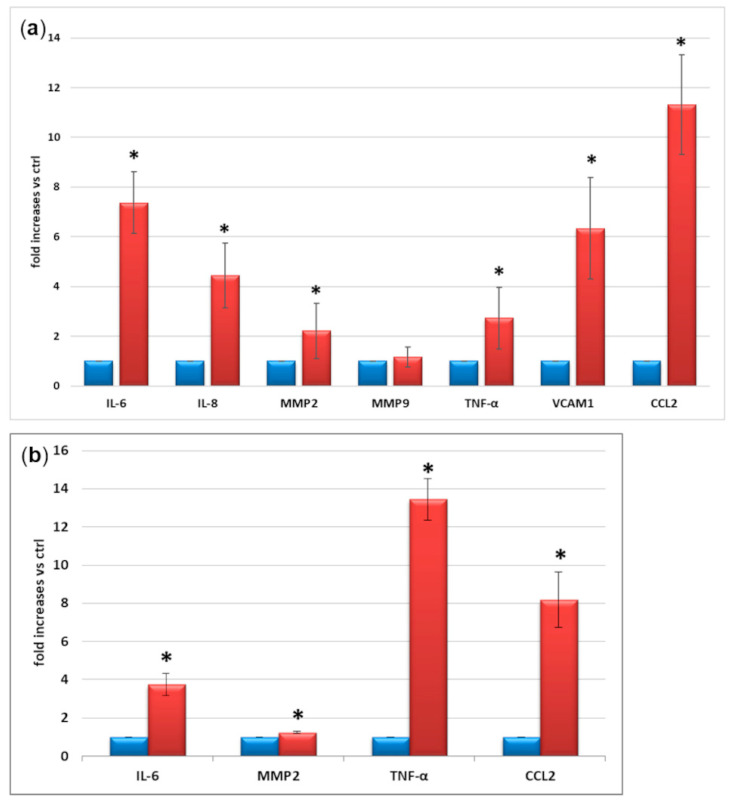
(**a**) mRNA expression and (**b**) secretion in the supernatants of inflammatory markers in control conditions (blue) and after M1 activation (red). M1 activation was achieved by exposing microglial cells for 24 h to TNFα 10 ng/mL + IL1β 20 ng/mL + IFNγ 50 ng/mL. Mean of five experiments ± SD, * *p* < 0.05.

The expression of a panel of well-known inflammatory factors was measured by RT-PCR, in control conditions and following 24 h M1 stimulation. As shown in Figure 4a, we found dramatic increases in IL-6 (>7-fold), interleukin-8 (IL-8) (>4-fold), matrix metalloproteinase-2 (MMP2) (2-fold), TNF-α (2.5-fold), vascular cell adhesion molecule 1 (VCAM1) (6-fold), and CCL2 (>11-fold) in M1 cells in comparison with control. These results were validated measuring the secretion in the supernatants of the most relevant cytokines (Figure 4b). We confirmed an increased release of IL-6 (4-fold), MMP2 (1.2-fold), TNF-α (13-fold), and CCL2 (8-fold) from M1 cells in comparison with control.

### 2.5. Morphological and Functional Changes

**Figure 5:**
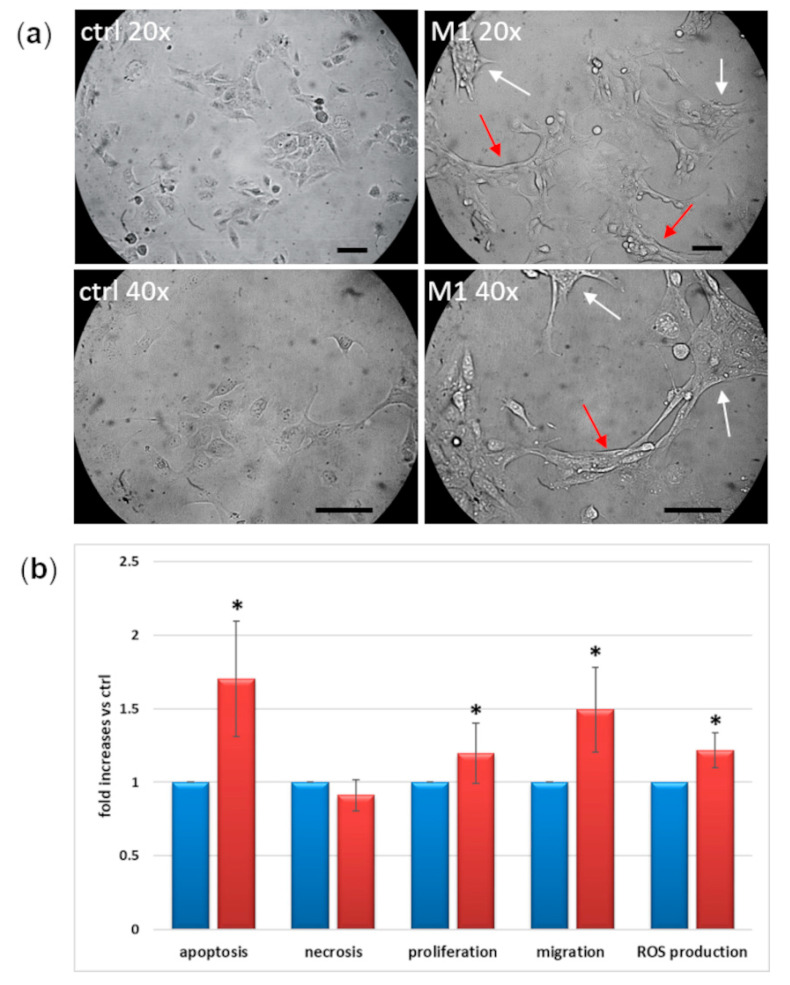
Functional changes of IM-HM cells after 24 h M1 exposure: (**a**) morphological aspect: comparison between physiological conditions (ctrl) and M1. M1 cells assume an amoeboid shape (white arrows) and form tubular structures (red arrows), characteristic of activated cells. Images were taken at 20x or 40x objective magnification, and scale bar indicates 50 μm; (**b**) apoptosis, necrosis, proliferation, migration, and reactive oxygen species (ROS) production in control (blue) and M1-activated (red) cells. M1 activation was achieved by exposing microglial cells for 24 h to TNFα 10 ng/mL + IL1β 20 ng/mL + IFNγ 50 ng/mL. Mean of five experiments ± SD, * *p* < 0.05.

Figure 5 shows morphological (a) and functional changes (b) in IM-HM cells following 24 h exposure to M1 stimulation. M1 cells assume an amoeboid shape and form tubular structures, which are characteristic of activated cells (Figure 5a). Compared with control conditions, in M1 conditions they show +70% apoptosis rate, 12% increase in proliferation, and +20% ROS production. Most of all, their migration rate is increased by +50% (Figure 5b).

**Figure 6:**
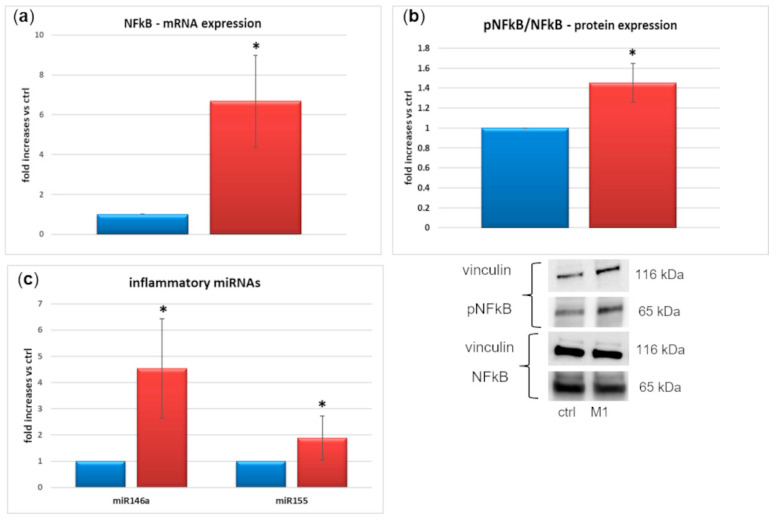
(**a**) Nuclear factor kappa B (NFkB) mRNA expression, mean of five experiments ± SD, * *p* < 0.05; (**b**) phospo-NFkB (pNFkB)/NFkB protein expression, mean of five experiments ± SD, * *p* < 0.05, plus representative images of one relevant Western blotting (WB) experiment; (**c**) inflammatory miRNAs 146a and 155 expression, mean of five experiments ±SD, * *p* < 0.05. Blue = control cells, red = M1-activated cells. M1 activation was achieved by exposing microglial cells for 24 h to TNFα 10 ng/mL + IL1β 20 ng/mL + IFNγ 50 ng/mL. Uncropped WB are shown in Supplementary Material Figure S1.

We also found a consistent increase (+65%) in nuclear factor kappa B (NFkB) mRNA expression (Figure 6a) and a +45% phospho-NFkB/total NFkB protein expression in M1-activated cells (Figure 6b). Finally, the expression of well-known pro-inflammatory miRNAs was 4.5-fold (miR146a) and 1.9-fold (miR155) increased in IM-HM cells following M1 activation (Figure 6c).

## 3. Discussion

In this paper we characterize and describe the susceptibility to inflammatory stimuli of a new commercially available immortalized line of human microglial cells and its potential suitability as a tool to study the complex interactions between the neuronal and vascular components of the retina in health and in DR.

Inflammation mediated by the microglia plays, in fact, a primary role in the cascade of events leading to DR. Microglial cells can be regarded as the resident macrophages inside the CNS and the retina, where they patrol and control the microenvironment through the release of immune factors. The microglia can assume two different phenotypes: pro-inflammatory M1, and anti-inflammatory M2. Usually, microglial cells are in the M2 state, showing several long ramifications. In the case of inflammation, which must be seen as a defensive mechanism towards harmful stimuli, they shift to the M1 phenotype, characterized by an amoeboid shape, retraction of cytoplasm processes, high mobility [20], and release of pro-inflammatory cytokines [6]. After the elimination of dangerous molecules, microglial cells downregulate the release of pro-inflammatory molecules and, eventually, revert to the M2 state, involving the discharge of anti-inflammatory factors [5]. During chronic diseases, such as DR, characterized by prolonged inflammation and hyperglycemia, this delicate equilibrium may be impaired and microglial cells remain in the activated state [2] in response to the continuous stimulus, worsening inflammation [6]. The release of inflammatory molecules from the microglia, in turn, also affects the microvascular components of the retina, mediating the release of pro-inflammatory and apoptotic factors by retinal pericytes while inhibiting the expression of pro-survival proteins [13] and, ultimately, leading to vascular leakage and angiogenesis.

All this considered, microglial cells may play a pivotal role in the pathogenesis of DR, and deeper studies of their interactions with pericytes and endothelial cells are needed. Unfortunately, human microglial cells are difficult to obtain, and most studies still use murine-derived cells. However, animal cells can behave differently from human ones, as we have demonstrated comparing bovine and human retinal pericytes [18,19]. From this perspective, we purchased and characterized IM-HM cells. These cells grow rapidly in the appropriate culture conditions, show a characteristic triangular/polygonal shape with several cytoplasmic processes in physiological conditions (M2 anti-inflammatory state), and express typical microglial markers (Iba-1, CD14, CD16, and CD68) [21]. They are also positive for TMEM119, capable of discriminating between resident microglia and peripheral macrophages [14], and CD11b, which is typical of human-derived cells [15]. These morphology and marker expressions are superimposable with those of other human microglial lines described in the literature [14,15,16]. It is worth noticing that the human microglial cell line HMC3, which was established and characterized in 1995 [17], was later found to be negative for the expression of CD68 and CD11b [22], suggesting that some of the original characteristics might have been lost in time [23].

Subsequently, we verified the response of IM-HM cells to inflammatory stimuli, trying at first to induce M1 activation by exposing them to various concentrations of LPS for different times and checking the release of NO in the supernatant by the Griess reaction. This is the most commonly used method in the literature. However, we could detect a release of nitric oxide in none of the conditions tested. Lack of NO release by human microglia has already been described [24,25], underlining once again the difference between human and murine cells and the need for species-specific cell models. Consistently, protein expression of pro-inflammatory (iNOS and IFN-γ) and anti-inflammatory (TGF-β) markers was analyzed to countercheck this finding, showing no differences between LPS-treated and untreated cells. This led us to hypothesize that, similarly to what was described by other authors [26,27], human microglial cells are not susceptible to LPS-stimulation, marking a difference with commonly used murine microglial cells, like BV-2 [2,13,28]. According to van der Poel and co-workers [27], tolerance to microbial stimuli may constitute a sort of protection to prevent inflammation-derived neuronal injury.

After excluding LPS as an inflammatory inducer, we tested several combinations of the pro-inflammatory cytokines TNF-α, IL-1β, IL-6, and IFN-γ on microglial cells. These cytokines are commonly used to induce microglia M1 activation, with synergistic effects [15,29,30], and are increased in the vitreous of diabetic patients with DR [11,31,32]. We evaluated their effect on the expression of IFN-γ and MHC class I, as markers of microglial activation, and TGF-β (anti-inflammatory factor). Finally, we found that a cocktail of TNF-α, IL-1β, and IFN-γ exerted the maximum effect at the lowest concentrations and used it for the subsequent evaluations. It is worth noticing that, while pro-inflammatory IFN-γ and MHC I increased, and anti-inflammatory TGF-β, IL-10, and arginase 1 decreased, following 24 h exposure to the M1 cytokine cocktail, NO release remained undetectable in all supernatants, and no difference in iNOS expression between untreated and treated cells was found in any of the conditions tested. This confirms our previous data on LPS stimulation and is consistent with previous evidence [24,25,33], postulating the different induction of iNOS in mouse vs. human microglia. Moreover, it may also account for the lack of release of NO by human microglia. Even though cells in the CNS express very low levels of MHC proteins, stimulation with IFN-γ increases the gene expression of several members of MHC, including MHC class I, by the microglia [25,34]. In inflammatory conditions, activated microglia can also produce IFN-γ [35]. On the other hand, IL-10 loss is known to promote a microglial shift to the M1 phenotype [36] by up-regulating IL-8 [37], while the effects of arginase 1 are still controversial [38]. In murine models, TGF-β1 is essential for postnatal microglia maturation and for microglia homeostasis and activation in adults. Its reduction is associated with neurological disorders associated with abnormal microglia activation, such as Parkinson’s and Alzheimer’s diseases [39]. Enhanced expression and increased secretion of a panel of pro-inflammatory factors (IL-6, IL-8, MMP2, TNF-α, VCAM1 and CCL2), known to be released by microglial cells in response to inflammatory and hyperglycemic stimuli [2,6,7,8,9,28,40], and to be present in the retinal microenvironment in patients with DR [41], was also detected in microglial cells stimulated with the M1 cocktail. The expression and release of these pro-inflammatory factors is consistent with what happens both in rodent [7,8,9,13,28] and in other human microglial cell lines [14,15,16,23].

Following M1 stimulation, IM-HM cells change their morphology, assuming an amoeboid shape, with retraction of cytoplasmic processes and subsequent formation of tubular structures, consistent with the description of M1-activated microglia [15,20]. Moreover, activated IM-HM cells proliferate and migrate more than resting M2 cells, these also being characteristic features of M1 microglia [11,42]. Increased proliferation was also described as a response to IL-1β stimulation [11], and microglial cell number was markedly increased in retinas from subjects with DR [43]. At the same time, we found quite surprisingly that M1-stimulated IM-HM cells are more subjected to apoptosis. Evidence in the literature shows that apoptosis is not only programmed cell death, but may also play key roles in tissue regeneration; this equilibrium between surviving and dying cells was called “compensatory proliferation” and is triggered by signals released from apoptotic cells [44]. Ping et al. [45] demonstrated a simultaneous increase of proliferation and apoptosis in vascular smooth muscle cells, hypothesizing that this may accelerate atherosclerosis in diabetic mice. As regards the microglia, Askew et al. [46] showed that, in adult mouse and human brain, microglia has a high proliferation rate coupled to apoptosis, resulting in a highly regulated control of microglial cell numbers maintained throughout life, both in healthy and unhealthy neural tissue. Another study interpreted this phenomenon as an auto-regulatory mechanism to eliminate excess activated cells [47]. On the other hand, necrosis is an uncontrolled cell death, often due to external physical or chemical damages. Our cells do not undergo necrosis after M1 activation, and this may indicate that the inflammatory stimulus does not simply destroy the cells, but induces a more “physiological” response.

Oxidative stress is a hallmark of DR. Hyperglycemia triggers both the direct and the indirect production of ROS through the increase of several metabolic pathways involved in glucose damage, resulting also in the accumulation of AGEs, superoxide overproduction, and increased NF-κB production [48]. ROS induce NF-κB phosphorylation and translocation to the nucleus [6,7,9], where it fosters activation of the microglia and the expression of inflammatory cytokines [6,7,8,9,11,12]. Interactions between AGEs and their receptors (RAGEs) are also able to induce microglia switch to the M1 phenotype [49]. Activated microglia enhances the oxidative damage inside the retina, by producing, in turn, ROS [9]. In murine microglial BV-2 cells, 24–48 h high glucose exposure increased microglia proliferation, secretion of pro-inflammatory factors, and iNOS [12], while a study on the human microglial cell line HMC3 [50] showed that the M1 switch with release of cytokines in response to high glucose stimulation was a rather late event (96 h), indicating a different response of human vs. murine cells. Our present data, consistently with others in the literature [13,14,15,16], demonstrate that microglia activation induced by pro-inflammatory insults occurs already after 24 h. In this study, we did not measure the direct effects of high glucose exposure on IM-HM, because our primary goal was to verify their M1 activation in response to inflammatory stimuli, in order to assess this cell line for further studies on the neurovascular unit. However, consistently with the above-mentioned findings, we demonstrate that 24 h IM-HM exposure to the M1 cocktail increases ROS production, NF-kB expression and activation, and cytokine expression and release. Thus, we can argue that inflammation and hyperglycemia exert similar effects on human microglial cells, but the inflammatory insult is more immediate. The release of ROS, inflammatory and apoptotic mediators from activated microglia, because of the exposure to combined inflammation and hyperglycemia, exerts toxic effects on surrounding tissues, especially the microvasculature [13].

Finally, we show increased expression of miR-155 and miR-146a following M1 induction in human microglial cells. These are two well-known pro-inflammatory miRNAs, closely linked to inflammatory mediators whose up-regulation we discussed above. In fact, their induction is NF-kB-dependent [51,52] and miR-146a increase was described in human primary neuronal-glial co-cultures and microglial cells as a consequence of stimulation by ROS and pro-inflammatory cytokines (IL-1b and TNF-α) [53,54,55]. While miR-155 mediates the propagation of inflammation, with downregulation of anti-inflammatory molecules, miR-146a is responsible for modulating its amplitude [55].

## 4. Conclusions

Diabetic retinopathy has long been considered a microvascular disease, and for decades in vitro research has focused on the two cell types that constitute the capillary vessel wall, endothelial cells, and pericytes. More recently, a role for neurodegeneration in the early development of DR was postulated. The interest of neuroscientists for pericytes is growing, because, due to their localization, they constitute a sort of bridge between the vascular and neural sides of the retina and play a key role in blood–brain/blood–retinal barrier maintenance [13,56,57]. Therefore, we believe that, nowadays, in vitro studies on DR cannot leave out of consideration actors belonging to both the neuroretina and the microvasculature. Our results show that the IM-HM microglial cell line may constitute a valuable tool for the study of the cascade of events leading to DR, if stimulated with suitable molecules able to induce a pro-inflammatory activation. In perspective, more complex co-culture models involving cells of the retinal microvasculature (human pericytes and retinal endothelial cells), together with microglia, will help understand the multifaceted interactions of the neurovascular unit in humans with a species-specific approach.

## 5. Materials and Methods

### 5.1. Cell Culture

Immortalized human microglial cells (IM-HM) were purchased from Innoprot (Bizkaia, Spain), stored, and thawed following manufacturer’s instructions. They were seeded in type I collagen-coated T75 flasks, at a concentration of 10,000 cell/cm^2^ in Microglia medium (Innoprot, Bizkaia, Spain), reaching confluence approximately after 72 h. IM-HM cells between passages 4–10 were used for experiments.

### 5.2. M1 Activation

To select the best treatment to induce M1 (pro-inflammatory) switch, we tested several combinations of well-known pro-inflammatory molecules at different time points. Their concentrations were chosen in accordance with previous reports in the literature [14,15,16,28]. Microglial cells were seeded in 6-well plates at a concentration of 50,000 cells/well and cultured for 24 h in their medium. They were then switched to DMEM without FCS and exposed to pro-inflammatory stimuli. First, we tried LPS at different concentrations (100/200/500/1000 ng/mL) and times of exposure (1, 2, and 4 days), and measured NO release in the supernatants by the Griess reaction. We also measured by Western blotting the expression of the pro-inflammatory iNOS and IFN-γ, and anti-inflammatory TGF-β, in cell lysates. Untreated cells maintained in DMEM without FCS were used as controls. Subsequently, we tested various combinations of TNF-α, IL-1β, IL-6, and IFN-γ, and checked their effects on IFN-γ, MHC class I, and TGF-β expression in cell lysates. Details are listed in Supplementary Material Table S1.

We finally chose a 24 h exposure to 10 ng/mL TNF-α + 20 ng/mL IL-1β + 50 ng/mL IFN-γ to induce M1 activation in IM-HM cells for the subsequent experiments.

All reagents were purchased from Merck (Darmstadt, Germany).

### 5.3. Griess Reaction

Nitrix oxide (NO) release in the supernatants was determined through the Griess Reagent Kit for Nitrite Determination (Merck, Darmstadt, Germany), according to the manufacturer’s instructions.

### 5.4. Western Blot Analysis

IM-HM cells were lysed using M-PER Mammalian Protein extraction reagent (Thermo Fisher Scientific, Waltham, MA, USA) added with 10 µL/mL protease inhibitor cocktail kit (Thermo Fisher Scientific, Waltham, MA, USA), to extract total proteins. Extracts were kept ice-cold and cleared by centrifugation at 20,000× *g* for 15 min at 4 °C. The supernatant was aliquoted and stored at −80 °C. Protein content was measured through the Bradford reaction.

Thirty micrograms of proteins were loaded on pre-cast gels (4–20% Mini-PROTEAN^®^ TGX™ Precast Gel, Bio-Rad, Hercules, CA, USA), separated by electrophoresis, and transferred to nitrocellulose membranes. Immunoblotting was performed by incubating the membranes with relevant antibodies (Supplementary Material Table S2). Immunoreactive bands were visualized using the enhanced chemiluminescence (ECL) Western blotting protocol (Merck, Darmstadt, Germany). The relative signal strength was quantified by densitometric analysis (1D Image Analysis System, Kodak, Rochester, NY, USA), and values normalized against vinculin.

### 5.5. Expression of Microglial Markers

The expression of microglial markers was measured by FACS analysis using Guava easyCyte™ Flow Cytometer (Merck-Millipore, Darmstadt, Germany), after 48 h from thawing. Cells were washed twice with PBS and detached by Cell Detachment Factor (Merck, Darmstadt, Germany). After spinning, 1 × 10^6^ cells were resuspended in 100 µL ice-cold PBS + 1% BSA + relevant antibody, incubated for 1 h at 4 °C, and washed twice. In the case of unconjugated primary antibodies (Iba1, TMEM119), cells were incubated with the relevant secondary Ab (anti-rabbit FITC or anti-mouse PE) for 1 h at 4 °C and washed twice. Labeled cells were resuspended in 200 µL PBS + 1% BSA and examined by FACS. Antibodies used are detailed in Supplementary Material Table S2.

### 5.6. Quantitative Real Time PCR (qRT-PCR)

Total RNA was extracted by HighPure RNA Isolation kit (Merck, Darmstadt, Germany), and 200 ng of RNA were reverse-transcribed using High Capacity cDNA Reverse Transcription Kits (Thermo Fisher Scientific, Waltham, MA, USA). qRT-PCR was performed by 48-well StepOne Real Time System (Applied Biosystems, Waltham, MA, USA) using Power SYBR™ Green PCR Master Mix (Thermo Fisher Scientific, Waltham, MA, USA). Relative gene expression was determined using the 2^-ΔΔCT^ method and normalized against β-actin. Primers used are listed in Supplementary Material Table S2.

### 5.7. Cytokine Secretion

Secretion in the supernatants of relevant cytokines was measured by ELISA, using human IL-6 ELISA kit (Invitrogen, Waltham, MA, USA, cat. n. KAC1261), MMP2 Human ELISA Kit (Invitrogen, Waltham, MA, USA, cat. n. KHC3081), Human TNF-α ELISA Kit (Invitrogen, Waltham, MA, USA, cat. n. KAC1751), and Human MCP-1 (CCL2) Platinum ELISA (Affymetrix eBioscience, Waltham, MA, USA), according to the manufacturers’ instructions.

### 5.8. Cell Function Parameters

IM-HM proliferation was measured as DNA synthesis through the Cell Proliferation ELISA BrdU kit (Merck, Darmstadt, Germany), and apoptosis and necrosis using the Cell Death Detection ELISA^PLUS^ kit (Merck, Darmstadt, Germany), according to the manufacturer’s instructions.

Cell migration rate was assayed using the colorimetric QCM Chemotaxis Cell Migration Assay (Merck, Darmstadt, Germany), according to the instructions. Briefly, cells were seeded inside 8 µm pore polycarbonate membranes and exposed to M1-inducing conditions. After 24 h, cells that were still inside the insert were removed and those migrated through the membrane stained. The stain was subsequently extracted and transferred to a 96-well ELISA plate for colorimetric reading at 560 nm.

Reactive oxygen species (ROS) production was evaluated by exposing cells in 96-well/plates to 25 µmol/L H2DCFDA (Invitrogen, Waltham, MA, USA) in medium without phenol red and FCS, for 45′ at 37 °C. Wells were then washed and added with fresh medium. Fluorescence was measured at 490 nm excitation/520 nm emission at different time-points.

### 5.9. miRNAs

Total RNA was extracted from IM-HM cells using mirVana RNA isolation kit (Thermo Fisher Scientific, Waltham, MA, USA), which also allows for the isolation of small RNAs, according to the manufacturer’s instructions. RNA was quantified spectrophotometrically (Nanodrop ND-1000, Thermo Scientific, Waltham, MA, USA), and 200 ng of RNA were reverse-transcribed using miScript Reverse Transcription Kit (Qiagen, Hilden, Germany). qRT-PCR was performed by 48-well StepOne Real Time System (Applied Biosystems, Waltham, MA, USA) using a miScript SYBR Green PCR Kit (Qiagen, Hilden, Germany). Specific primers to miR146a and miR155 (Supplementary Material Table S2) were used. miRNA expression was normalized against the small nuclear RNA RNU6B.

### 5.10. Statistical Analysis

Results are mean ± SD of five independent experiments, normalized against control (IM-HM cells in physiological conditions). Statistical comparisons were carried out by two-tailed Student’s t-test for paired data. Results were considered significant for *p* ≤ 0.05. SPSS software version 26.0 (IBM, Armonk, NY, USA) was used for statistical analysis.

## Data Availability

Data will be made available by the corresponding author upon reasonable request.

## Supplementary Materials

The following supporting information can be downloaded at: https://www.mdpi.com/article/10.3390/ijms23105745/s1.
