# Supplementary material for: Characterization of an Immortalized Human Microglial Cell Line as a Tool for the Study of Diabetic Retinopathy
Source: Int J Mol Sci. 2022 May 20;23(10):5745. doi: 10.3390/ijms23105745 (PMC9145666; doi:10.3390/ijms23105745)

Characterization of an immortalized human microglial cell line as a tool for the study of diabetic retinopathy

Aurora Mazzeo, Massimo Porta, Elena Beltramo

Supplementary Material

Table S1. Factors and combinations tested to determine the best mixture to induce M1 activation in microglial cells. All factors are of human origin, concentrations and exposure times were chosen according to the literature. Nitric oxide (NO) release was measured in the supernatants through Griess reaction, inducible nitric oxide synthase (iNOS), interferon-γ (IFN-γ), MHC I (pro-inflammatory markers), and transforming growth factor-β (TGF-β, anti-inflammatory) by Western blotting in cell lysates. We chose the combination that exerted the maximum effects with the lowest concentrations: 10 ng/mL tumor necrosis factor –α (TNF-α) + 20 ng/mL interleukin 1β (IL-1β) + 50 ng/mL IFN-γ. Lypopolisaccharide (LPS) had no effect on IM-HM cells. Legend: no = non-detectable, ns = no difference vs ctrl (unexposed cells), ↑ = low increase vs ctrl (<25%), ↑↑ = medium increase vs ctrl (25-50%), ↑↑↑ = high increase vs ctrl (50-75%), ↓= decrease (<25%) vs ctrl, - = not tested.

Factors	Concentrations (ng/mL)	Time (h)	NO release	iNOS	IFNγ	MHC II	TGFβ
**LPS**	100/200/500/1000	24/48/96	no	ns	ns	-	ns
**TNFα**	10/20	24/48/96	no	ns	ns	↑↑	ns
**IL6**	10/20	24/48/96	no	ns	ns	ns	ns
**IL1β**	20/40	24/48/96	no	ns	↑	↑↑	↓
**IFNγ**	20/50	24/48/96	no	ns	ns	↑	↓
**IL1β+IL6**	20+20 / 40+20	24	no	ns	↑	-	ns
**TNFα+IL1β**	10+20 / 10+40	24	no	ns	↑	↑↑	ns
**TNFα+IL6**	10+20	24	no	ns	↑	-	ns
**TNFα+IL1β+IL6**	10+20+20 / 10+40+20	24	no	ns	↑	-	↓
**TNFα+IL1β+IL6+LPS**	10+20+20+500	24	no	ns	↑	-	↓
**IL1β+IFNγ**	20+20 / 20+50	24	no	ns	↑	↑↑	↓
**TNFα+IFNγ**	10+50	24	no	ns	↑	↑↑	ns
**TNFα+IL1β+IFNγ**	10+40+50	24	no	ns	↑↑	↑↑↑	↓
**TNFα+IL1β+IFNγ**	**10+20+50**	24	no	ns	↑↑	↑↑↑	↓

Table S2. List of primers/antibodies used and relative working dilutions. Primers were all purchased from Eurofins Genomics.

Human miRNA primers	
miR-155	TTAATGCTAATCGTGATAGGGGT
miR-146a	TGAGAACTGAATTCCATGGGTT

Human mRNA primers	*forward*	*reverse*
IL-6	AGACAGCCACTCACCTCTTCAG	TTCTGCCAGTGCCTCTTTGCTG
IL-8	AAGAGAGCTCTGTCTGGACC	GATATTCTCTTGGCCCTTGG
MMP2	AGCGAGTGGATGCCGCCTTTAA	CATTCCAGGCATCTGCGATGAG
MMP9	CTGAGTCAGCACTTGCCTGTCA	TATGCTTACCCCAGAACCCTCCAAT
TNFα	CTCTTCTGCCTGCTGCACTTTG	ATGGGCTACAGGCTTGTCACTC
VCAM1	CTTTTGGAGTCGAAGATGAGGAAA	CACTACTATCGCAAAACTGACTGAA
CCL2	CCACTTATCACTCATGGAAGATCCC	GAGTAACTGCGCTGAGTGTGTT
NF-κB (p65)	AGGCAAGGAATAATGCTGTCCTG	ATCATTCTCTAGTGTCTGGTTGG

WB antibodies	*Producer*	*Cat. #*	*RRID*	*Dilution*
MHC class I	Santa Cruz Biotechnology	sc-25619	AB_649123	1:1000
NF-κB (p65)	Abcam	ab31481	AB_2300947	1:1000
NF-κB (p65) (phospho-S536)	Abcam	ab131109	AB_11160495	1:1000
IFN-γ	Santa Cruz Biotechnology	sc-52556	AB_629704	1:1000
IL-10	Santa Cruz Biotechnology	sc-8438	AB_627793	1:1000
Vinculin	Merck	V9131	AB_477629	1:1000
TGFβ	Santa Cruz Biotechnology	sc-374659	AB_10988781	1:500
Arg1	Santa Cruz Biotechnology	sc-20150	AB_2058955	1:1000
iNOS	Cell Signaling	20609		1:1000

Flow cytometry antibodies	*Producer*	*Cat. #*	*RRID*	*Dilution*
CD14 PE	Miltenyi Biotec	130-110-519	AB_2655051	1:50
CD68 FITC	Miltenyi Biotec	130-096-964	AB_2659042	1:20
CD64 PE	Thermo Fisher Scientific	12-0649-42	AB_10667885	1:20
CD11B PE	Merck	FCMAB178P	AB_10806342	1:50
Iba1	Thermo Fisher Scientific	PA5-27436	AB_2544912	1:50
CD16 FITC	Miltenyi Biotec	130-113-954	AB_2726427	1:20
TMEM119	Atlas Antibodies	AMAb91528	AB_2797214	1:20

Secondary antibodies	*Producer*	*Cat. #*	*RRID*	*Dilution*
Anti-rabbit FITC	Abcam	ab6717	AB_955238	1:1000
Anti-mouse PE	Merck	P9287	AB_261243	1:1000

Isotype (negative) controls	*Producer*	*Cat. #*	*RRID*	*Dilution*
FITC Mouse IgG1	BD Biosciences	558905	AB_397156	1:50
PE Mouse IgG1	BD Biosciences	558904	AB_397155	1:50

Figure S1. Uncropped WB related to Figure 6b.

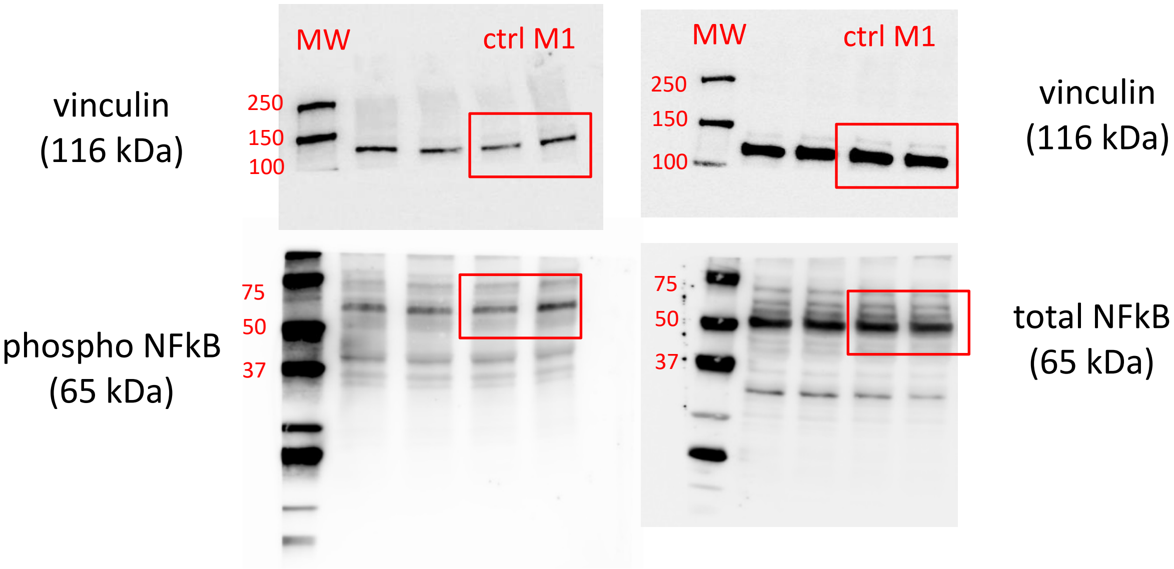

Supplement: Supplementary file 1 [file ijms-23-05745-s001.zip › ijms-1700496-supplementary.pdf]
